# Autophagy as a Therapeutic Target in Diabetic Nephropathy

**DOI:** 10.1155/2012/628978

**Published:** 2011-10-19

**Authors:** Yuki Tanaka, Shinji Kume, Munehiro Kitada, Keizo Kanasaki, Takashi Uzu, Hiroshi Maegawa, Daisuke Koya

**Affiliations:** ^1^Department of Medicine, Shiga University of Medical Science, Otsu, Shiga 520-2192, Japan; ^2^Division of Diabetes & Endocrinology, Kanazawa Medical University, Kahoku-Gun, Ishikawa 920-0293, Japan

## Abstract

Diabetic nephropathy is a serious complication of diabetes mellitus, and its prevalence has been increasing worldwide. Therefore, there is an urgent need to identify a new therapeutic target to prevent diabetic nephropathy. Autophagy is a major catabolic pathway involved in degrading and recycling macromolecules and damaged organelles to maintain intracellular homeostasis. The study of autophagy in mammalian systems is advancing rapidly and has revealed that it is involved in the pathogenesis of various metabolic or age-related diseases. The functional role of autophagy in the kidneys is also currently under intense investigation although, until recently, evidence showing the involvement of autophagy in the pathogenesis of diabetic nephropathy has been limited. We provide a systematic review of autophagy and discuss the therapeutic potential of autophagy in diabetic nephropathy to help future investigations in this field.

## 1. Introduction

The prevalence of diabetes mellitus has been increasing worldwide during recent years, and this is estimated to continue in the future [1, 2]. Diabetic nephropathy is a serious complication of diabetes mellitus and is the most common cause of end-stage renal disease [3, 4]. The increasing prevalence of diabetes mellitus and its complications, including diabetic nephropathy, has therefore become a major health problem worldwide. There is now an urgent need to identify new therapeutic target molecules or cellular processes that underlie the pathogenesis of diabetic nephropathy to establish an additional therapeutic option.

 Hyperglycemia-mediated alteration of extra- and intracellular metabolism, such as advanced glycation end products [5], increased protein kinase C activity [6], and abnormal polyol metabolism [7], has been recognized as classical pathogenesis of diabetic nephropathy. In addition, intracellular stress associated with renal hypoxia [8, 9], mitochondrial reactive oxygen species (ROS) [10–13], and endoplasmic reticulum (ER) stress [14–16] has recently been proposed and focused as new pathogenesis of diabetic nephropathy. Thus, to maintain the cellular homeostasis against stress condition derived from organelle dysfunction or hypoxia may be a new therapeutic target of diabetic nephropathy.

**Figure 1:**
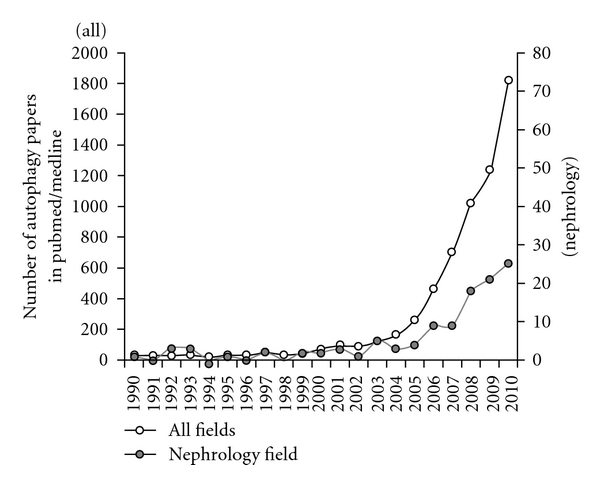
*Substantial increases in the number of autophagy-related papers indexed in PubMed/Medline.* The number of autophagy-related papers in all fields has increased remarkably over recent decades. Corresponding with the increase of autophagy-related papers in all fields, publication of autophagy-related papers in nephrology fields, also gradually increased.

Autophagy, a lysosomal protein degradation pathway in cells, plays a crucial role in removing protein aggregates as well as damaged or excess organelles to maintain intracellular homeostasis and cell integrity [17]. It has recently been highlighted because it can be stimulated by multiple types of cellular stressors including starvation, hypoxia, or ER stress. The study of autophagy in mammalian systems and in disease states is advancing rapidly, and many investigators are entering this new and exciting field (Figure 1). It has been revealed that autophagy plays a crucial role in several organs, especially in metabolic organs, and that its alteration is involved in the pathogenesis of metabolic [18–21] and age-related diseases [22–27]. The functional role of autophagy in the kidneys is currently under intense investigation (Figure 1), and it has been revealed that autophagy has a renoprotective role in several animal models including those used for aging and acute kidney injury [26–31]. However, the role of autophagy in diabetic nephropathy remains unclear.

Alteration of several nutrient-sensing pathways is related to the development of metabolic diseases, such as type 2 diabetes and its vascular complications. Major nutrient-sensing pathway involves the mammalian target of rapamycin (mTOR) [32–35], AMP-activated protein kinase (AMPK), [36–40] and oxidized NAD- (NAD^+^-) dependent histone deacetylase (Sirt1) [41–43], which are also recognized as the regulatory factors of autophagy under nutrient-depleted condition. As described above, autophagy can be induced by intracellular stresses that are involved in the pathogenesis of diabetic nephropathy [44]. Thus, alteration of these nutrient-sensing pathways under diabetic condition may impair the autophagic stress response stimulated by intracellular stress, which may lead to exacerbation of organelle dysfunction and subsequent diabetic nephropathy. 

The above findings lead us to hypothesize that autophagy is involved in the pathogenesis of diabetic nephropathy and is a potential therapeutic option. Therefore, we provide a systematic review of autophagy and discuss its therapeutic potency in diabetic nephropathy to help future investigations in this field.

## 2. Autophagy

The term autophagy is derived from Greek and means self-eating. It is highly conserved from yeast to mammals and is a bulk degradation process that is involved in the clearance of long-lived proteins and organelles. Autophagy has two major roles in cells: to recycle intracellular energy resources in response to nutrient-depleted conditions and to remove cytotoxic proteins and organelles under stressful conditions. Autophagy works to maintain cell homeostasis under various stressful conditions. Several types of autophagy have been recognized in cells: macroautophagy, microautophagy, and chaperone-mediated autophagy; these differ in their mechanisms and functions [45, 46]. Of these three types of autophagy, macroautophagy is most prevalent and hereafter is referred to as autophagy. In this paper, we focus on the mechanisms and functions of autophagy.

## 3. Molecular Mechanisms of Autophagy

**Figure 2:**
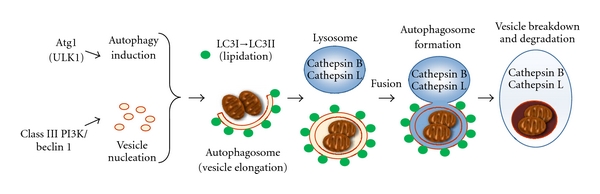
*Scheme of autophagic pathways.* Autophagic pathways consist of four steps: initiation, nucleation, elongation, and closure. Autophagy is initiated by the nucleation of an isolation membrane (phagophore). The phagophore elongates and closes on itself to form an autophagosome. Fusion of an autophagosome with a lysosome forms an autolysosome and, where the acid hydrolases in the lysosome, breaks down the inner membrane and cytoplasmic contents.

During macroautophagy, *de novo* isolation membranes (phagophores) elongate and fuse while engulfing a portion of the cytoplasm within double-membraned vesicles (autophagosomes) (Figure 2). Several origins of autophagosomes have been reported, including the ER [47–49], mitochondria [50], and plasma membrane [51]. Four major steps are involved in the formation of autophagosomes: initiation, nucleation, elongation, and closure. During these steps, autophagy-related proteins are involved (Figure 2). Autophagy is initiated by the unc-51-like kinase (Ulk) 1 (mammalian ortholog of the yeast autophagy-related genes (Atg)1) complex, which is composed of Ulk1 Ser/Thr protein kinase, Atg13, and FIP200 (mammalian homolog of the yeast Atg17) [52–54]. The phosphorylation of Atg13 and FIP200 by Ulk1 is essential for triggering autophagy. Phagophore nucleation is dependent on Beclin 1 (Atg6 in yeast)—an hVps34 or class III phosphatidylinositol 3-kinase (PI3K) complex, which consists of hVps34, hVps15, Beclin 1, and Atg14 [55, 56].

During autophagosome elongation/closure, two dependent ubiquitin-like conjugation systems are involved: Atg12 and LC3 (the mammalian ortholog of the yeast Atg8) [57]. The Atg12-Atg5 conjugate, which forms the Atg12-Atg5-Atg16 complex, contributes to the stimulation and localization of the LC3 conjugation reaction. The cytosolic isoform of LC3 (LC3-I) is conjugated to phosphatidylethanolamine through two consecutive ubiquitination-like reactions that are catalyzed by E1-like enzyme Atg7 and the E2-like enzyme Atg3 to form LC3-II [58]. Thus, LC3-II formation is recognized as a marker of existence of autophagosomes in cell or animal experiments [59–61]. After formation, the autophagosomes merge with the lysosomal compartment to form autolysosomes. The protein p62, also known as sequestosome 1 (SQSTM1), is known to localize to autophagosomes via LC3 interaction and to be constantly degraded by the autophagy-lysosome system [62, 63]. The accumulation of p62 is observed in autophagy-deficient cells [62, 63].

## 4. Methods Available for Monitoring Autophagy

It is necessary to keep in mind several important points as we monitor and assess the autophagy activity to prevent misconceptions. Some reviews about the methods for autophagy research have been published [59–61]. As briefly below summarized, some methods including electron microscopy (EM), detection of endogenous LC3 or green fluorescent protein (GFP)-LC3 by fluorescence microscopy, and detection of LC3-II by Western blotting are useful in monitoring the number of autophagosomes. However, these methods have some limitations. An accumulation of autophagosomes does not always mean increased formation of autophagosomes and may represent inhibited maturation of autolysosomes (or amphisomes). Simply counting the number of autophagosomes is insufficient for assessing autophagy activity. Autophagy flux is a term that represents a serial process of autophagy, including the synthesis of autophagosomes, the delivery of cargo to lysosomes, and the degradation of autolysosomes. To distinguish whether the accumulation of autophagosomes is caused by induction of autophagy or inhibition of autophagosome maturation and/or degradation of autophagic substrates in the lysosome, and then assess autophagy activity, an autophagy flux assay is more reliable than counting the number of autophagosomes. There are some useful assays to monitor autophagy flux. These include the LC3 turnover assay, or measurement of total levels of autophagic substrates such as LC3, GFP-LC3, and p62. Furthermore, several types of autophagy inhibitors and activators have recently become available to modulate the activity of autophagy processes. Pharmacological inhibitors of autophagy are PI3-kinase inhibitors such as wortmannin, LY294002, or 3-methladenine (3-MA) and inhibitors that block autophagosome-lysosome fusion or degradation of autophagic cargo in autolysosomes, such as E64d, pepstatin A, and bafilomycin A. However, a major problem is that there are no highly specific inhibitors or activators of autophagy. Thus, it is strongly recommended that pharmacological studies should be combined with studies that investigate deficiency/reduction of autophagy-related genes by genetic knockout/knockdown of ATG genes or dominant-negative mutant autophagy proteins, including Atg3, Atg5, Atg7, and Beclin 1.

## 5. Role of Nutrient Stress in Autophagy and Diabetic Nephropathy

**Figure 3:**
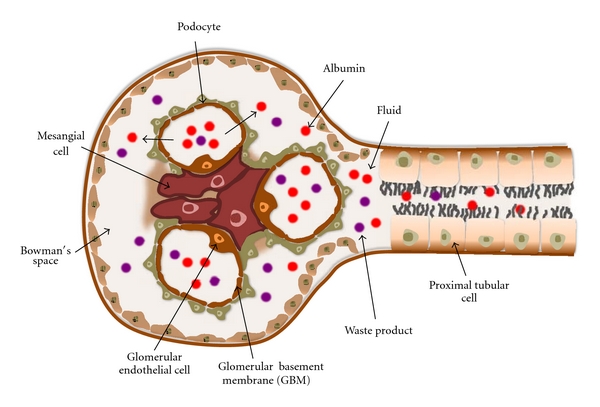
*Schematic representation of nephron.* The basic unit of the kidney is the nephron, which consists of a glomerulus and a series of tubules lined by a continuous layer of epithelial cells. The glomerulus consists of mesangial cells and a capillary wall with endothelial cells, glomerular basement membrane, and visceral epithelial cells (podocytes). Fluid containing albumins and waste products is filtered through glomerulus, enters urinary space, and is reabsorbed by the proximal tubular cells.

The kidney is a structurally complex organ and is essential in several functions including excretion of the waste products of metabolism, regulation of body fluid volume, maintenance of appropriate acid balance, and secretion of a variety of hormones. The basic unit of the kidney is the nephron, which consists of a glomerulus and a series of tubules lined by a continuous layer of epithelial cells (Figure 3). The glomerulus consists of mesangial cells and a capillary wall with endothelial cells, glomerular basement membrane, and visceral epithelial cells (podocytes) (Figure 3). Among them, since podocytes play essential role to maintain glomerular filtration barrier, podocyte injury leads to proteinuria and glomerulosclerosis, which are major features of diabetic nephropathy. Podocytes are terminally differentiated cells with a limited proliferative capacity. Therefore, the fate of podocyte depends on its ability to cope with stress. Excess fluid filtered through glomerulus enters urinary space and is reabsorbed by the proximal tubular cells (Figure 3). The proximal tubular cells serve as a system to degrade several molecules reabsorbed from urinary space. Thus, autophagy may be essential to maintain their homeostasis and functions in both podocytes and proximal tubular cells, which might be altered in diabetic condition. If autophagy system is altered in diabetic condition, this alteration of autophagy may be involved in the pathogenesis of diabetic nephropathy. 

As expected, autophagy has been identified in both podocytes and proximal tubular cells and is regulated by a variety of stimuli including nutrient stress. Nutrient depletion is the most potent physiological inducer of autophagy, among several that have been reported to regulate autophagy. Here, we show the roles of mTOR, AMPK, and Sirt1, in the regulation of autophagy. The alteration of these pathways is involved in the pathogenesis of several kidney diseases including diabetic nephropathy.

### 5.1. mTOR

Several studies have shown that hyperactivation of the mTOR pathway in diabetic nephropathy plays a pivotal role in the hypertrophy of existing glomerular and tubular cells [64, 65] and is associated with podocyte injury and the progressive decline of glomerular filtration rates. Other studies have suggested that inhibition of the mTORC1 pathway with rapamycin has renoprotective effects on the progression of diabetic nephropathy in models of type 1 [33] and type 2 diabetes [32, 66–69]. Some reports have shown the additive renoprotective effects of rapamycin treatment including prevention of mesangial expansion and glomerular membrane thickness in type 1 diabetic rats [33] and attenuation of increased glomerular expression of laminin-*β*1 protein in type 2 diabetic mice [32, 67]. It has been reported that activation of the mTOR pathway is involved in the increased expression of profibrotic cytokines, such as TGF-*β*1 and connective tissue growth factor, and subsequent interstitial fibrosis in diabetic nephropathy [32–34]. Furthermore, more recent reports have shown that mTORC1 activity is essential to maintain podocyte homeostasis, but its hyperactivation is a causes of glomerular lesion of both type 1 and type 2 diabetic nephropathy [68, 69]. Complete deletion of podocyte mTORC1 activity in podocyte-specific Raptor-deficient mice causes podocyte injury [68]. In contrast, podocyte-specific mTORC1 hyperactivation by podocyte-specific tuberous sclerosis complex (TSC) 1-knockout mice show podocyte injury and glomerular lesion similar to diabetic nephropathy [69]. Finally, podocyte-specific Raptor-heterozygous mice show partial deletion of mTORC1 activity in podocyte and resistance to the development of diabetic nephropathy in both STZ-induced type 1 diabetic mice [68] and type 2 diabetic* db/db* mice [69].

In addition to the above-mentioned function, inhibition of autophagy is a main role of mTOR pathway [44, 58, 70]. Among the several signaling pathways that regulate autophagy in mammalian cells, the classical pathway of serine/threonine kinase, mTOR, plays a major role in the negative regulation of autophagy because it integrates signals that are emitted by growth factors, amino acids, glucose, and energy status [71]. Autophagy is inhibited by the activation of TOR under hypernutrient conditions [44, 58]. The mTOR pathway involves two functional complexes: mTOR complex 1 (mTORC1) and mTORC2. mTORC1, which consists of the mTOR catalytic subunit, regulatory associated protein of mTOR (Raptor), G protein *β*-subunit-like protein (G*β*L), proline-rich Akt substrate of 40 kDa (PRAS40), and DEP domain-containing mTOR-interacting protein (Deptor) [72], is sensitive to the immunosuppressant rapamycin [73, 74]. This complex regulates cell growth, metabolism (by integrating amino acid and growth factor signals), energy, and oxygen status [75]. The mTORC1 complex suppresses autophagy via phosphorylation and inactivation of Ulk1, an initiator of autophagosome formation [76]. Although no direct evidences have been provided, hyperactivation of mTOR pathway may suppress autophagy in podocyte and tubular cells in diabetic condition. Furthermore, enhanced activity of autophagy may be involved in the renoprotective effects of rapamycin treatment in diabetic nephropathy. The mTORC2 complex is less sensitive to rapamycin and includes mTOR, rapamycin-insensitive companion of mTOR (Rictor), G*β*L, stress-activated protein kinase-interacting protein 1 (Sin1), protein observed with Rictor (PROTOR), and Deptor [75, 77]. The mTORC2 complex regulates cytoskeletal organization, metabolism, and cell survival [75, 78, 79]. However, until now, the role of mTORC2 in regulation of autophagy has remained unclear.

### 5.2. AMPK

AMPK is activated under energy-depleted conditions and is likely to be suppressed in diabetic nephropathy. It has been reported that AMPK is inactivated (decreased phosphorylation of AMPK) in glomeruli and tubules in both type 1 and type 2 diabetic animal models [40, 81–84], which are reversed by agents such as metformin and resveratrol along with attenuation of diabetic glomerular and tubular injury [40, 85, 86]. This introduces the question of how decreases in AMPK activity can be involved in the pathogenesis of diabetic nephropathy. In type 1 and 2 diabetic kidneys, intrarenal lipid metabolism is altered, which is characterized by enhanced renal lipogenesis and suppressed lipolysis [87–90]. AMPK-mediated phosphorylation inactivates a lipogenic enzyme, acetyl-CoA carboxylase, which results in decreased lipogenesis and enhanced lipolysis [91]. Decreases in renal AMPK activity in these mouse models may be a mechanism of altered renal lipid metabolism and subsequent lipotoxicity-associated renal damage. Since AMPK can affect various cellular metabolism as wells as lipid metabolism [92, 93], the other molecular mechanism should be involved in AMPK-mediated renoprotection.

AMPK plays a central role in the integration of several stress stimuli and is a positive regulator of autophagy in response to nutrient-depleted conditions. AMPK monitors the energy status of the cell by sensing its AMP/ATP ratio [93]. Several upstream kinases, including liver kinase B1 (LKB1), calcium/calmodulin kinase kinase (CaMKII) *β*, and TGF-*β*-activated kinase-1 (TAK1), can activate AMPK by phosphorylating a threonine residue on its catalytic *α* subunit [93]. AMPK can crosstalk with the mTORC1 signal during multiple steps of autophagy regulation. AMPK induces autophagy by inhibiting mTORC1 activity via phosphorylation of its regulatory-associated proteins [44, 58, 94]. Recent studies have shown that AMPK-dependent phosphorylation of Ulk1 induces autophagy [94, 95]. A balance between mTORC1 and AMPK likely directly regulates Ulk1 activity and subsequent autophagy initiation [44]. Thus, in addition to the above-mentioned mechanism, AMPK-mediated induction of autophagy may be involved in its renoprotection. AMPK activation may be linked to autophagy for the maintenance of renal homeostasis in diabetic kidney.

### 5.3. Sirt1

Sirtuins, the silent information regulator 2 family, were originally identified as NAD^+^-dependent deacetylases in experiments in lower species and consist of seven members, Sirt1–Sirt7, in mammals [96, 97]. Sirtuins have been identified as antiaging molecules under calorie-restricted conditions and environmental stress. Some mammalian sirtuins, especially Sirt1, have been shown to play important roles in the regulation of aging, or in the pathogenesis of age-related metabolic diseases such as type 2 diabetes [41, 42, 96]. An increase in the intracellular concentration of NAD^+^ by caloric restriction can activate Sirt1. Results that demonstrate the role of Sirt1 in autophagy are still lacking compared with those for mTOR and AMPK, but they have recently been increasing. Sirt1 can deacetylate essential autophagic factors such as Atg5, Atg7, and LC3 [98] and has been shown to induce autophagy. Furthermore, Sirt1 deacetylates the transcription factor Forkhead box O3a (Foxo3a), which leads to enhanced expression of proautophagic BCL2/adenovirus E1V 19-kDa interacting protein 3 (Bnip3) [26].

Renal expression of Sirt1 decreases in type 1 diabetic animal models [99, 100]. Also, reduced forms of nicotinamide adenine dinucleotide (NADH) are metabolites of glucose and fatty acids. Thus, NAD^+^/NADH ratios are decreased in cells under conditions where nutrients are in excess, such as diabetes. Sirt1-deacetylase activity should decrease in diabetic nephropathy. Although direct renoprotective effects of Sirt1 in diabetic nephropathy have yet to be elucidated, Sirt1 has shown renoprotective activity in aging kidneys and fibrotic kidney diseases. The previously mentioned findings lead us to speculate that activation of Sirt1 should also have therapeutic efficacy in diabetic nephropathy. Furthermore, Sirt1-induced autophagy activation may contribute to Sirt1-mediated renoprotective effect in diabetic nephropathy.

## 6. Regulation of Autophagy by Intracellular Stress

Besides nutrient stress, autophagy is upregulated by several intracellular stresses, such as hypoxia, ROS, and ER stress [44]. Based on recent reports, this process is probably a compensatory response to maintain cell integrity. Furthermore, these intracellular stresses have recently been focused on as a pathogenesis of diabetic nephropathy, in addition to the classical pathogenesis of diabetic nephropathy.

### 6.1. Oxidative Stress

Under conditions where nutrients are in excess, such as diabetes and obesity, the production of ROS in the kidneys is enhanced by high glucose concentrations [101, 102]. Furthermore, high levels of free fatty acids, especially polysaturated fatty acids, also induce ROS production in the kidneys [88, 103]. Oxidative stress is a by-product of mitochondrial respiration and is associated with cell dysfunction. Actually, a recent report has shown abnormal mitochondrial morphology in diabetic kidney [95, 104], suggesting that diabetic kidney fails to remove damaged mitochondria. Thus, restoring the ability to control mitochondria homeostasis should be a therapeutic target of diabetic nephropathy.

Mitochondrial quality control is mediated by mitochondrial autophagy (mitophagy) [105]. Similarly, oxidative stress can induce autophagy to remove damaged mitochondria to protect cells. Thus, autophagy-mediated quality control of mitochondria and subsequent reduction of ROS should be essential to protect kidney in diabetic condition. It has been reported that exogenous hydrogen peroxide activates protein kinase RNA-like ER kinase (PERK), which subsequently phosphorylates eukaryotic initiation factor-2*α*, activates Atg4, and inhibits mTOR [106]. In response to cellular stress or damage, mitochondrial membranes can be permeabilized. The autophagic recognition of depolarized mitochondria is mediated by a refined voltage sensor, which involves the mitochondrial kinase, phosphatase, and tensin homolog-induced putative kinase 1.

### 6.2. Hypoxia

In early-stage diabetic nephropathy, hypoxia is aggravated by manifestations of chronic hyperglycemic abnormalities of red blood cells [107, 108], oxidative stress [109], and diabetes mellitus-induced tubular apoptosis; as such, tubulointerstitial hypoxia in diabetes mellitus might be an important early event.

Hypoxia is also a stimulatory factor of autophagy. Hypoxia-induced autophagy largely depends on hypoxia-inducible factor-1*α* (HIF-1*α*), which is a transcription factor that is activated and stabilized under hypoxic conditions [110, 111]. HIF-1*α* activates transcription of Bnip3 and Bnip3L and subsequently induces autophagy. Normally, Beclin 1 interacts with Bcl-2 proteins. Bnip3 can disrupt this interaction, liberating Beclin 1 from Bcl-2 in cells and leading to autophagy. Thus, HIF1*α*-induced Bnip3 overexpression promotes autophagy [112]. The transcription of Bnip3 is also upregulated by the transcription factor FOXO3, which is deacetylated and positively regulated by Sirt1 [26]. Hypoxia causes damage to the mitochondria and intracellular accumulation of ROS [113]. Removing the damaged mitochondria under hypoxic conditions is also an important role of Bnip3-mediated autophagy. Thus, to investigate whether hypoxia-induced and Sirt1-mediated autophagy is altered in diabetic kidney is interesting. If it is altered, to restore autophagy activity even under diabetic condition should be important to protect kidney form hypoxia.

### 6.3. ER Stress

ER stress has recently been focused as a pathogenesis of diabetic nephropathy. The induction of ER stress and subsequent apoptosis by hyperglycemia and high levels of free fatty acids (polysaturated fatty acids) are observed in podocytes [114]. Additionally, in proteinuric kidney diseases, including diabetic nephropathy, massive proteinuria filtered from glomeruli causes ER stress responses and subsequent apoptosis in renal tubular cells [14, 115]. Thus, to suppress inadequate ER stress is thought as a therapeutic strategy of diabetic nephropathy.

It is known that ER stress as well as hypoxia and ROS also cause autophagy. The ER is not only involved in protein synthesis and maturation but may also constitute a major source/scaffold for the autophagic isolation membrane [47]. When misfolded proteins are not exported efficiently to the cytoplasm and accumulate in the ER, the unfolded protein response (UPR) is often induced [116–118]. The UPR consists of three main branches that are controlled by the ER membrane proteins: PERK; activating transcription factor-6 (ATF6); inositol requiring enzyme 1 (IRE1) [116–118]. Among these UPR-related proteins, PERK and ATF6 have been reported to induce autophagy [44]. PERK induces the transcriptional activation of LC3 and Atg5 through the action of the transcription factors ATF4 and CCAAT-enhancer-binding protein homologous protein, respectively [119]. It has been suggested that IRE1 is also involved in the induction of autophagy by phosphorylation of Beclin 1 via c-Jun NH2-terminal kinase-1 [44]. Enhanced and prolonged ER stress causes several pathogenic features such as apoptosis and inflammation [117, 118]. Thus, autophagy-mediated ER degradation (ERphagy) may be required for cell protection from prolonged cytotoxic ER stress shown in diabetic kidney.

## 7. Autophagy in the Kidneys

The study of autophagy has previously been undertaken in lower species. The study of autophagy in mammalian systems is advancing rapidly and has revealed that mammalian autophagy is involved in the pathogenesis of various metabolic or age-related diseases [18–27].

Recently, nephrologists have also entered this exciting field of study. In this section, we review recent studies on the pathophysiology of autophagy in the kidneys. Autophagy has been observed in various parts of the kidneys, including proximal tubules, and thick ascending limbs. In particular, in podocytes, higher levels of constitutive autophagy have been observed using GFP-LC3 transgenic mice even under normal conditions [27]. As for the role of autophagy in renal pathophysiology, several researchers have reported the significance of autophagy in experimental renal injury models. In several experimental animal models of glomerulonephritis, including puromycin aminonucleoside and adriamycin-induced proteinuria, autophagy has been identified and shown to play renoprotective and antiproteinuric roles in podocytes through the use of podocyte-specific Atg5 knockout mice [27]. It has been recently reported that the normal aging process suppresses autophagy in podocytes, and that podocyte-specific deletion of *Atg5* leads to glomerulopathy in aging mice that is accompanied by accumulation of oxidized and ubiquitinated proteins, ER stress, and proteinuria [27].

In renal tubules as well as in podocytes, autophagy has been reported to play a renoprotective role under several pathological conditions. In renal ischemia-reperfusion injury models, the upregulation of autophagy to protect the kidneys was observed using 3-MA, chloroquine [28], and proximal tubular epithelial cell-specific Atg5 knockout mice [29]. Additionally, in cisplatin-induced acute kidney injury models, the increase of autophagosomes was observed by EM, LC3-II Western blotting [30], and GFP-LC3 transgenic mice [31]. Hypoxia is one of the causes of renal tubular damage in aged kidney [120]. We have previously shown that hypoxia-induced autophagy activity declined with age, which led to accumulations of damaged mitochondria and mitochondrial ROS in the kidney [26]. Interestingly, long-term calorie restriction (CR) restored autophagy activity even in aged kidney [26]. As a mechanism, Sirt1-mediated autophagy was essential in CR-mediated renoprotection in aged kidney [26]. Bnip3 expression is essential to induce autophagy under hypoxic condition [121] and is positively regulated by a transcriptional factor Foxo3a [122]. This regulation was altered in aged kidney. On the other hand, CR-mediated Sirt1 activation deacetylated and activated Foxo3a transcriptional activity and subsequent Bnip3-mediated autophagy even in aged kidney [26]. Furthermore, the kidney of heterozygous Sirt1-knockout mice showed lower autophagy activity along with the decrease in Bnip3 expression, and thus they were resistant to CR-mediated antiaging effects [26]. This finding suggests that Sirt1 is essential for CR-mediated renoprotection.

Thus, accumulative evidence has demonstrated the pathophysiological importance of autophagy in the kidneys (Table 1). However, the role and existence of autophagy in other types of renal cells besides podocytes and proximal tubular cells is not known.

## 8. Perspective

It is evident that the above-mentioned nutrient-sensing signals exist in the kidneys. However, what are their physiological roles in this organ? The kidneys require sufficient amounts of ATP for maintenance of their functions and avidly consume oxygen to drive mitochondrial oxidative phosphorylation among major organs. A small percentage of oxygen consumed by mitochondria is incompletely reduced to ROS, and this unremitting generation of oxidants during mitochondrial respiration, albeit in small amounts, may cumulatively damage the kidneys, which are heavily dependent on mitochondrial metabolism. Regulating mitochondrial metabolism in response to nutrient conditions via regulation of autophagy that can remove damaged mitochondria and subsequent ROS may be a physiological role of renal nutrient-sensing signals.

**Figure 4:**
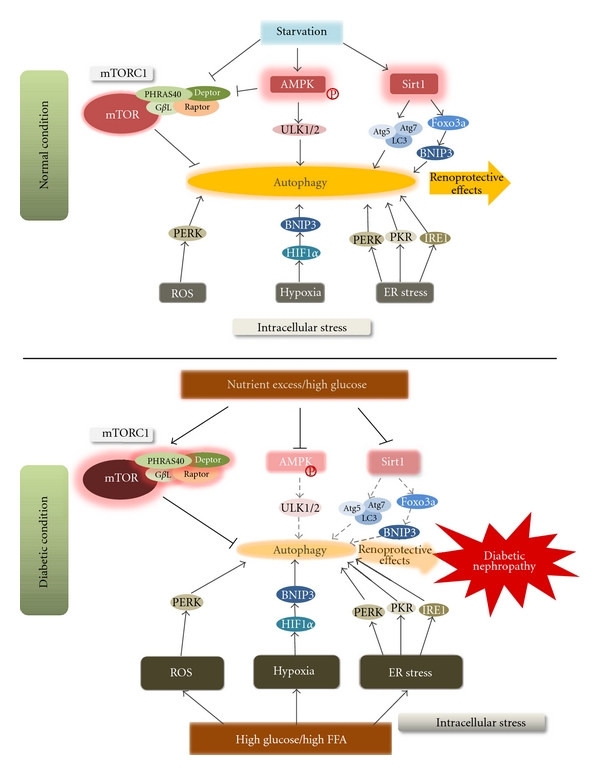
*Regulation of autophagy by nutrient and intracellular stresses and the relationship between autophagy and the progression of diabetic nephropathy.* Under normal conditions, intracellular stresses such as hypoxia, mitochondrial ROS, and ER stress induce autophagy. Nutrient depletion enhances autophagy by inhibiting mTORC1 and by activating AMPK and Sirt1. This activation of autophagy helps to maintain intracellular homeostasis and may have renoprotective effects. In contrast, under diabetic conditions, high glucose or FFA levels increase intracellular stresses, leading to the progression of diabetic nephropathy. Furthermore, nutrient excess and high glucose levels under diabetic conditions inhibit autophagy by inhibiting AMPK and Sirt1, and by activating mTORC1. This inactivation of autophagy may reinforce the progression of diabetic nephropathy. ROS: reactive oxygen species; ER: endoplasmic reticulum; mTORC1: mammalian target of rapamycin (mTOR) complex 1 (mTORC1); AMPK: AMP-activated protein kinase; FFA: free fatty acid.

Autophagy is regulated by nutrient conditions, and its alteration associates with various metabolic and age-associated diseases. Although studies on autophagy have methodological limitations, as outlined above, it is evident that autophagy deficiency is associated with podocyte and tubular cell injuries from the studies using Atg5-knockout mice [27, 29]. These findings lead us to hypothesize that autophagy is altered in diabetic kidneys, and autophagy deficiency should contribute to the pathogenesis of diabetic nephropathy. Altered nutrient-sensing signals in diabetic kidneys may contribute to accumulation of mitochondrial ROS via suppression of autophagy, which may be associated with initiation of the early stages of diabetic nephropathy. Both hypoxia and proteinuria-induced ER stress contribute to proximal tubular cell damage in the progressive and overt stages of diabetic nephropathy. Why do diabetic kidneys show a weakness against these stresses? How can we protect the kidneys from these stresses even under diabetic conditions? One answer may be derived from autophagy studies. Autophagy deficiency in diabetic kidneys may make tubular cells fragile under hypoxic and ER stress and possibly lead to progression of diabetic nephropathy (Figure 4). Activation of autophagy may be a therapeutic option for the advanced stages of diabetic nephropathy.

## 9. Concluding Comments

In recent decades, numerous investigators have been making efforts to identify the molecular mechanisms involved in the initiation and progression of diabetic nephropathy to develop new therapeutic strategies. However, end-stage renal disease due to diabetic nephropathy continues to increase worldwide. There is an urgent need to identify additional new therapeutic targets for prevention of diabetic nephropathy. We have provided a perspective on whether autophagy is involved in the pathogenesis of diabetic nephropathy and whether it is an acceptable new therapeutic target. Unfortunately, there have still not been many studies that have focused on autophagy in diabetic nephropathy. In the next few years, studies using Atg-gene knockout/knockdown mice combined with different methodologies will elucidate this possibility. Finally, these studies will ultimately give us a clearer perspective as to whether autophagy should be considered as a novel therapeutic target to halt the progression of diabetic nephropathy.

## Figures and Tables

**Table 1:** Autophagy-related kidney diseases.

Species and methods to monitor autophagy	Disease model	Effects of autophagy	Reference
Sprague-Dawley rats, immunohistochemistry of LC3 and Western blotting of LC3-II	Cyclosporine A-induced nephrotoxicity	Protection against tubular cell death	[80]

C57BL/6 mice, EM, and Western blotting of LC3-II	Cisplatin injury	Protection against tubular cell death	[30]

C57BL/6 mice, EM, immunofluorescence of LC3, and Western blotting of LC3-II	Aging	Protection against aging and hypoxia-related tubular damage	[26]

GFP-LC3 mice	Cisplatin injury	Protection against tubular cell death	[31]

C57BL/6 mice, EM, and Western blotting of LC3-II with 3-MA and chloroquine	Ischemia reperfusion	Protection against tubular cell death	[28]

Proximal tubular epithelial cell-specific Atg5-deficient mice	Ischemia reperfusion	Protection against tubular cell death	[29]

Podocyte-specific Atg5-deficient mice	Aging, protein overload-, LPS-, PAN-, and adriamycin-induced glomerular injury	Protection against podocyte injury	[27]

EM: electron microscopy; GFP: green fluorescent protein; 3-MA: 3-methladenine; Atg: autophagy-related genes; LPS: lipopolysaccharide; PAN: puromycin aminonucleoside.
